# Adenovirus vector-based incorporation of a photo-cross-linkable amino acid into proteins in human primary cells and cancerous cell lines

**DOI:** 10.1038/srep36946

**Published:** 2016-11-11

**Authors:** Ayami Kita, Nobumasa Hino, Sakiko Higashi, Kohji Hirota, Ryohei Narumi, Jun Adachi, Kazuaki Takafuji, Kenji Ishimoto, Yoshiaki Okada, Kensaku Sakamoto, Takeshi Tomonaga, Seiji Takashima, Hiroyuki Mizuguchi, Takefumi Doi

**Affiliations:** 1 Laboratory of Molecular Medicine, Graduate School of Pharmaceutical Sciences, Osaka University, 1-6 Yamadaoka, Suita, Osaka 565-0871, Japan; 2 Laboratory of Proteome Research, National Institute of Biomedical Innovation, Health and Nutrition, 7-6-8 Saito-Asagi, Ibaraki, Osaka 567-0085, Japan; 3 Center for Medical Research and Education, Graduate School of Medicine, Osaka University, 2-2 Yamadaoka, Suita, Osaka 565-0871, Japan; 4 Division of Structural and Synthetic Biology, RIKEN Center for Life Science Technologies, 1-7-22 Suehiro-cho, Tsurumi, Yokohama 230-0045, Japan; 5 Molecular Network Control Project, RIKEN Center for Life Science Technologies, 1-7-22 Suehiro-cho, Tsurumi, Yokohama 230-0045, Japan; 6 Department of Medical Biochemistry, Graduate School of Medicine, Osaka University, 2-2 Yamadaoka, Suita, Osaka 565-0871, Japan; 7 Japan Science and Technology Agency-Core Research for Evolutional Science and Technology (CREST), Kawaguchi, Saitama 332-0012, Japan; 8 Laboratory of Biochemistry and Molecular Biology, Graduate School of Pharmaceutical Sciences, Osaka University, 1-6 Yamadaoka, Suita, Osaka 565-0871, Japan

## Abstract

The site-specific incorporation of cross-linkable designer amino acids into proteins is useful for covalently bonding protein complexes upon exposure to light. This technology can be used to study networks of protein-protein interactions in living cells; however, to date it has only been applicable for use with a narrow range of cell types, due to the limited availability of plasmid-based transfection protocols. In the present study, we achieved adenovirus-based expression of a variant of an archaeal pyrrolysyl-tRNA synthetase and UAG-recognising tRNA pair, which was used to incorporate unnatural amino acids into proteins at sites defined by in-frame UAG codons within genes. As such, the site-specific photo-cross-linking method is now applicable to a wide variety of mammalian cells. In addition, we repositioned the reactive substituent of a useful photo-cross-linker*, N*^ε^-(*para*-trifluoromethyl-diazirinyl-benzyloxycarbonyl)-l-lysine (pTmdZLys), to the *meta* position, which improved its availability at low concentration. Finally, we successfully applied this system to analyse the formation of a protein complex in response to a growth signal in human cancerous cells and human umbilical vein endothelial cells. This adenovirus-based system, together with the newly designed cross-linkable amino acid, will facilitate studies on molecular interactions in various cell lines of medical interest.

The differential expression of cell proteins creates various networks of molecular interactions that are cell-type specific. Although co-immunoprecipitation is a facile and widely used method for analysing protein-protein interactions, it does not distinguish between direct and indirect interactions or between the actual interactions in cells and those falsely occurring in cell lysates. In addition, weakly bound proteins easily dissociate from each other during the purification process^1^. Photo-cross-linking methods can circumvent these drawbacks by covalently linking directly bound proteins when cells are exposed to light^2^,^3^,^4^.

The site-specific incorporation of photo-cross-linkable amino acids into proteins has enabled detailed analyses of protein-protein interactions in living cells, since the site-specificity allows for the identification of molecules that are bound to defined places within a protein^5^,^6^,^7^,^8^,^9^. For example, when *para*-benzoyl-l-phenylalanine (*p*Bpa) or *para*-trifluoromethyl-diazirinyl-l-phenylalanine (tmdPhe) was incorporated into the Src homology 2 (SH2) domain of GRB2, the molecules binding to this domain were identified separately from those binding to its SH3 domain^5^,^6^. Cross-linkable amino acids have been incorporated into proteins in mammalian cells by assigning them to the UAG stop codon. Two types of exogenous molecules are necessary for this expansion of the genetic code: UAG-reading tRNA and an engineered aminoacyl-tRNA synthetase (aaRS) that is able to attach specific unnatural amino acids to the tRNA molecule^10^,^11^,^12^. This tRNA–aaRS pair does not interact with endogenous tRNA or aaRS; therefore, cross-linkable amino acids are incorporated only at sites defined by the UAG codon. Despite the potential usefulness of the site-specific photo-cross-linking method, the required expression of exogenous molecules via plasmid-based transfection has limited its utility to a narrow range of cell types.

In the present study, we achieved the adenovirus (Ad) vector-based incorporation of cross-linkable amino acids into proteins in mammalian cells. The Ad vector has advantages over lentivirus and baculovirus vectors, which have both been previously used for expanding the genetic code^13^,^14^. First, various types of cells can be transduced by Ad with high efficiency, regardless of whether cells are proliferating or in a quiescent state^15^,^16^,^17^,^18^,^19^,^20^. Co-transduction by multiple Ad vectors is also highly efficient. Secondly, Ad has a relatively large cargo capability (up to 8-kb of extra DNA), and recombinant Ad is constructed easily by *in vitro* ligation or recombination and then amplified in “packaging” mammalian cell lines. Finally, Ad shows a high physicochemical stability in CsCl density gradient centrifugation, which allows for easy viral condensation. We used an *N*^ε^-benzyloxycarbonyl-l-lysine (ZLys) derivative as a photo-cross-linkable amino acid, because it has reactive groups at the end of a long side chain, which is advantageous in cross-linking^8^.

## Results and Discussion

### Developing the expression system for tRNA^Pyl^

**Figure 1:**
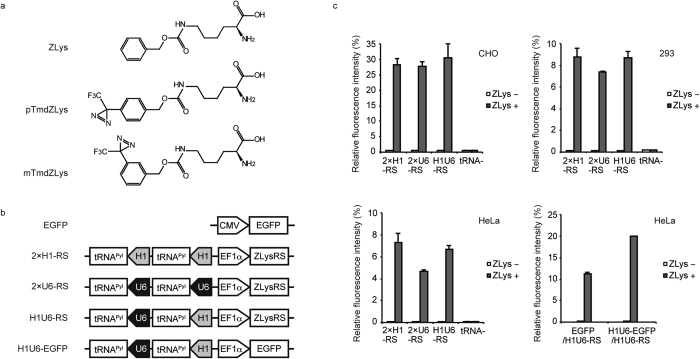
Incorporation of ZLys by different gene constructs for pyrrolysine tRNA (tRNA^Pyl^). (**a**) The chemical structures of ZLys, pTmdZLys, and mTmdZLys. (**b**) Schematic representations of the gene constructs for expressing tRNA^Pyl^. (**c**) Relative fluorescent intensities of EGFP produced under the indicated conditions. The intensities are shown with standard errors (n = 3).

ZLys and *N*^ε^-(*p*-trifluoromethyl-diazirinyl-benzyloxycarbonyl)-l-lysine (pTmdZLys), a cross-linkable derivative of ZLys (Fig. 1a), have been incorporated into proteins by using the same variant of pyrrolysyl-tRNA synthetase (PylRS), derived from *Methanosarcina mazei*, together with its cognate tRNA^Pyl^, which reads the UAG codon^8^,^21^. This variant is designated as ZLysRS. A U-to-C base substitution was incorporated into this tRNA to improve its ability to translate UAG^22^. Since the incorporation efficiency for unnatural amino acids largely depends on the efficiency of UAG-reading tRNA expression^10^, we tested human H1 and U6 promoters of RNA polymerase III, individually and in combination, for expressing tRNA^Pyl^. This tRNA lacks the internal promoter sequence recognised by the eukaryotic RNA polymerase III.

We created two gene constructs (2 × H1-RS, 2 × U6-RS) to express tRNA^Pyl^ from the H1 and U6 promoters, respectively, together with ZLysRS from the EF1α promoter (Fig. 1b). The U6 terminator was used to terminate the transcription of the tRNA genes. Each construct included two copies of the promoter-tRNA-terminator sequence in the same direction. We also constructed H1U6-RS, which included two tRNA genes under the control of the two different promoters, respectively (Fig. 1b). These three constructs were introduced into Chinese hamster ovarian (CHO), HEK293 c18, and HeLa cells using a plasmid vector. A mutant gene of enhanced green fluorescent protein (EGFP), EGFP(E18UAG), which has UAG in place of a Glu codon at position 18, was also introduced as a reporter. The fluorescence of EGFP was detected for all of the combinations between the gene constructs and cell types when growth media were supplemented with ZLys (Fig. 1c). These observations support that ZLys was incorporated at the UAG position, which allowed for the synthesis of full-length EGFP. The incorporation efficiency was estimated by comparing the fluorescence of the amber mutant EGFP gene to that of the wild-type gene with no in-frame UAG, and was found to vary largely with cell type (30% in CHO cells versus 7% to 8% in 293 c18 and HeLa cells). By contrast, the variation in efficiency was small among the different gene constructs, although 2 × U6-RS yielded lower efficiencies than the other two. The choice of H1U6-RS, rather than 2 × H1-RS and 2 × U6-RS, may be the better option to avoid possible recombination between sequences during viral genome replication^13^,^23^. Also, another advantage to using the hybrid gene construct for expressing tRNA^Pyl^, which is expected to work well regardless of cell type, is because the H1 promoter may work better than U6 in some cell lines, while U6 may work better in others.

To duplicate tRNA genes and enhance their expression, the tRNA genes from H1U6-RS were also included in the EGFP(E18UAG)-expressing plasmid, thereby creating H1U6-EGFP(E18UAG) (Fig. 1b). When H1U6-RS was paired with this plasmid instead of the original EGFP-expressing plasmid, the fluorescence yield was almost double (lower right panel, Fig. 1c), indicating that the multiplication of tRNA genes effectively improved the incorporation efficiency of unnatural amino acids.

### A photo-reactive amino acid that is useful at low concentrations

**Figure 2:**
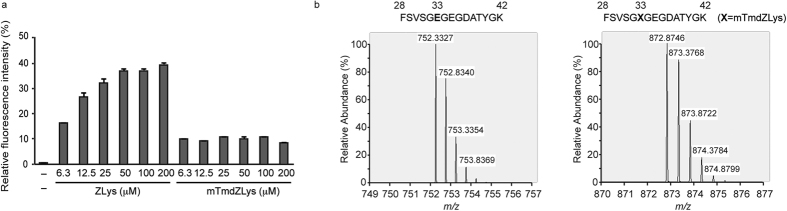
Site-specific incorporation of mTmdZLys into proteins. (**a**) Relative fluorescent intensities of EGFP produced with ZLys and mTmdZLys supplementation in the growth media at the indicated concentrations. The intensities are shown with standard errors (n = 3). (**b**) Mass-to-charge spectra of the trypsin-digested peptides (positions 28–42) from wild-type EGFP (left panel) and the product of its mutant gene with UAG at position 33 (right panel).

The cross-linkable pTmdZLys has been incorporated into proteins by using the ZLysRS-tRNA^Pyl^ pair, and its incorporation efficiency was significantly lower than that of ZLys^8^. We inferred that the bulky diazirinyl group at the *para* position of the benzene moiety of pTmdZLys causes a steric hindrance in the amino-acid binding pocket of ZLysRS, and that repositioning the reactive substituent might alleviate this problem. We tried to incorporate *N*^ε^-(*m*-trifluoromethyl-diazirinyl-benzyloxycarbonyl)-l-lysine (mTmdZLys) (Fig. 1a) into proteins with the same experimental settings (HEK293 c18 cells and the pOriP plasmid as the gene carrier) as those previously used for incorporating pTmdZLys^8^. The fluorescence of EGFP was detected when growth medium was supplemented with mTmdZLys at a concentration range of 6.25–200 μM (Fig. 2a), suggesting that ZLysRS-tRNA can incorporate mTmdZLys in response to UAG.

We performed mass spectrometric analyses of the tryptic digests of wild-type EGFP and its mutant containing mTmdZLys at the UAG position. The resulting peptides corresponded to positions 28–42 of the wild-type and mutant proteins, and the observed mass-to-charge (*m*/*z*) values were 752.3327 and 872.8746, respectively, which are almost identical to the theoretical *m/z* values of their doubly-charged ions (752.3335 and 872.8712, respectively) (Fig. 2b). Together with the observation that EGFP was synthesised only in the presence of mTmdZLys, these data strongly suggest that the amino acid was site-specifically incorporated at the UAG position.

Based on relative fluorescence intensities, the yields of EGFP with mTmdZLys were estimated to be 10% of that of EGFP expressed from the wild-type gene with no in-frame UAG. An increase in the concentration of mTmdZLys did not facilitate its incorporation into EGFP, whereas an elevation in the concentration improved the incorporation efficiency for ZLys (Fig. 2a) and pTmdZLys^8^. However, mTmdZLys was incorporated into EGFP at a remarkably low concentration (6.25 μM). By contrast, the incorporation efficiency of pTmdZLys was reportedly 4% at a concentration of 50 μM^8^. In most cases, unnatural amino acids are supplemented in the growth medium at a concentration ranging from 0.1 to 1 mM. Since a lower concentration of a reactive amino acid in the growth medium is desirable for avoiding adverse effects, mTmdZLys is preferable to pTmdZLys based on our results.

### Ad vector-based incorporation of ZLys

**Figure 3:**
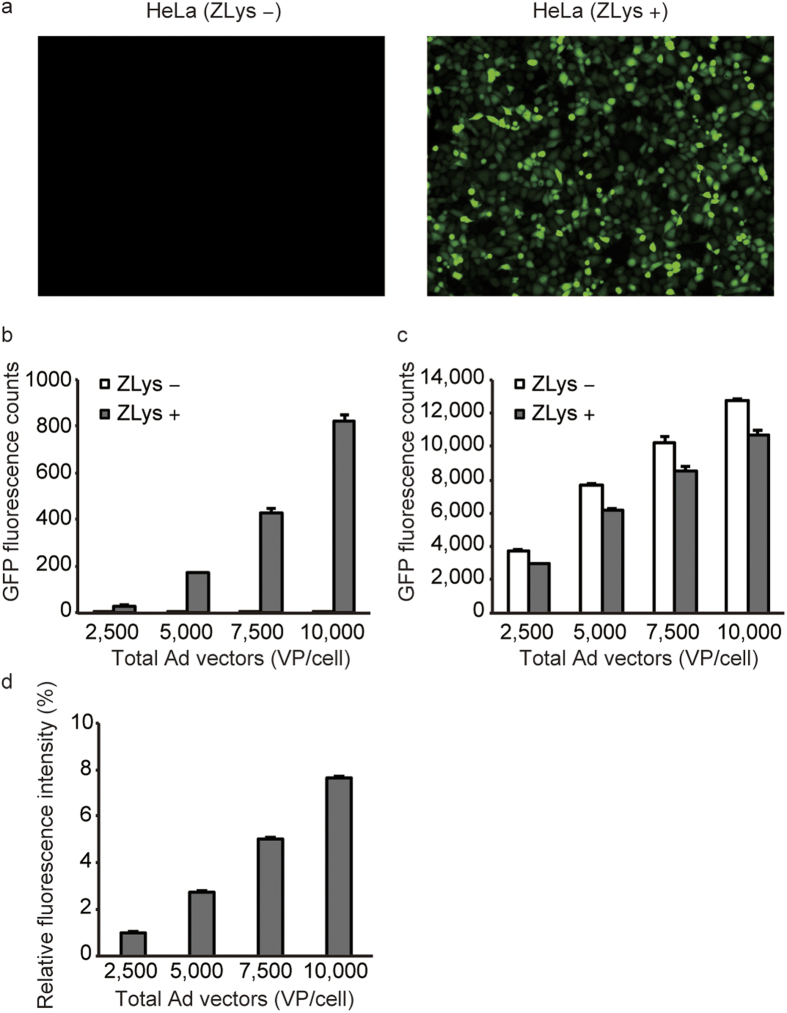
Adenovirus (Ad) -based incorporation of ZLys into EGFP. (**a**) Fluorescence images of the HeLa cells infected with the Ad vector encoding H1U6-EGFP(E18UAG) at a total VP/cell of 10,000 in the absence and presence of ZLys. (**b**) Fluorescence counts at VP/cell values from 2,500 to 10,000. (**c**) Fluorescence counts obtained when H1U6-EGFP(WT) was introduced in place of H1U6-EGFP(E18UAG) at the indicated VP/cell values. (**d**) Relative intensities of EGFP(E18UAG) to EGFR(WT) at the indicated VP/cell values. The mean intensities are shown with standard errors (n = 3).

We created Ad carrying the H1U6-EGFP(E18UAG) and H1U6-RS fragments, respectively, and infected HeLa cells with equal doses of the created viruses. The detection of fluorescence in the presence of ZLys suggests that the ZLysRS-tRNA^Pyl^ pair was successfully expressed in the cells, together with the EGFP UAG mutant (Fig. 3a). The intensity of fluorescence increased as the number of the applied viral particles per cell (VP/cell) of the Ad was increased from 2,500 to 10,000 (Fig. 3b). For comparison, the EGFP(E18UAG) gene in H1U6-EGFP(E18UAG) was replaced with the wild-type gene with no in-frame UAG. The fluorescence also increased in accordance with an increase in the VP/cell value from 2,500 to 10,000 (Fig. 3c), and the relative yields for EGFP(E18UAG) were calculated using these values as is shown in Fig. 3d. The maximal incorporation efficiency (8%) was obtained at a VP/cell value of 10,000.

**Figure 4:**
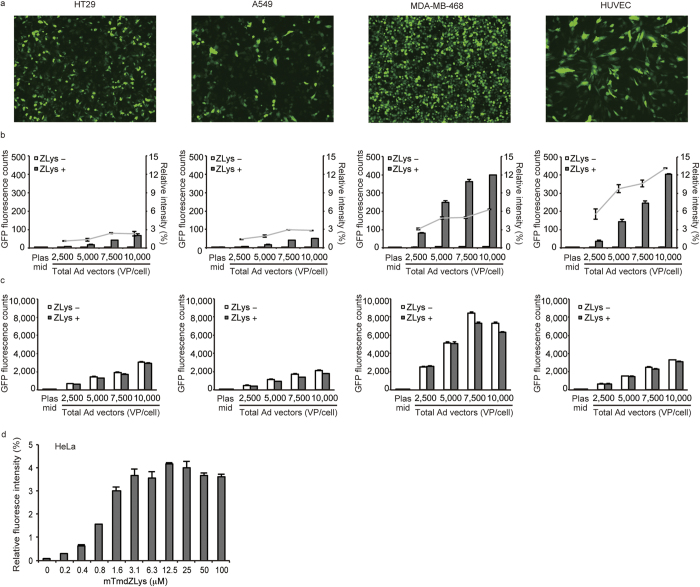
Adenovirus (Ad)-based incorporation of ZLys and mTmdZLys into EGFP in various cells. (**a**) Fluorescence images of the indicated cell types infected with Ad at a total VP/cell of 10,000 in the presence of ZLys. (**b**) Counts of ZLys-dependent fluorescence (bar graphs referring to the left axis) and relative fluorescent intensities (line graphs referring to the right axis) at the indicated VP/cell values in the cell types indicated at the top of the column. (**c**) Fluorescence counts of EGFP(WT) in the cell types indicated at the top of the column, in the absence and presence of ZLys and at the indicated VP/cell values. (**d**) The Ad-based incorporation of mTmdZLys into EGFP in HeLa cells at the indicated concentrations of the amino acid supplemented in the growth media. The fluorescent intensities are shown with standard errors (n = 3).

Next, we examined the applicability of the Ad system to a variety of cell types, including human tumour cell lines (A549, HT29, and MDA-MB-468) and primary cells (human umbilical vein endothelial cells, HUVEC), which are not suitable for plasmid-based transfection. Fluorescence was observed in all the cells infected with the Ad encoding H1U6-EGFP(E18UAG) and H1U6-RS (Fig. 4a and bar graphs in Fig. 4b). The fluorescence yield was higher than that obtained by plasmid transfection, demonstrating the superiority of Ad vectors for gene delivery to these cells (Fig. 4b). The efficiency of ZLys incorporation was estimated by comparing the fluorescence intensities of cells infected with H1U6-EGFP(WT) and those infected with H1U6-EGFP(E18UAG) (Fig. 4c). It was found that the incorporation efficiency largely varies with cell types. A549 yielded the lowest value (less than 3%), while HUVEC yielded the highest efficiency (about 12%) (Fig. 4b, line graphs). In every cell line, the expression level of EGFP(E18UAG) in the presence of ZLys was the highest at a VP/cell value of 10,000. The observed large difference in incorporation efficiency between cell types can probably be ascribed to their different infection susceptibilities for Ad^24^.

Finally, we tested the Ad-based incorporation of mTmdZLys into EGFP in HeLa cells. The fluorescence of the protein was detected over a wide range of concentrations of mTmdZLys in the growth medium. The intensity of fluorescence increased as the concentration was raised from 0.2 to 3.1 μM, whereas higher concentrations did not enhance the incorporation of mTmdZLys (Fig. 4d), which was consistent with the previous observation.

### Photo-cross-linking protein complexes involved in cell signalling in human cell lines

**Figure 5:**
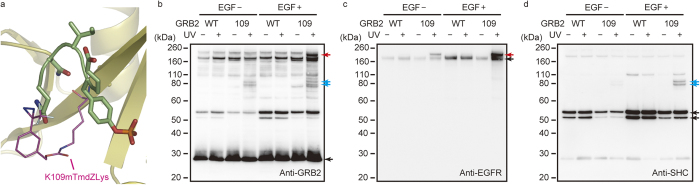
Photo-cross-linking between GRB2(mTmdZLys109) and endogenous cell-signalling proteins in human MDA-MB-468 cells. (**a**) A structural model of mTmdZLys (magenta sticks) in place of Lys109 of GRB2 (ribbons) bound to a tyrosine-phosphorylated peptide derived from EGFR (green sticks); the model was constructed by modifying the crystal structure data obtained from Protein Data Bank (code 1ZFP). (**b**–**d**) Western-blot analyses of cross-linked products. GRB2 proteins, tagged with FLAG peptide, were precipitated from protein fractions of the human cells treated as indicated. Then, western blotting was performed using anti-GRB2 antibody (**b**), anti-EGFR antibody (**c**), and anti-SHC antibody (**d**). Black arrows indicate GBR2 (**b**), EGFR (**c**), and SHC (**d**). Red and blue arrows indicate probable cross-linked complexes of GRB2 with EGFR and SHC, respectively.

By utilising the Ad-based system for incorporating mTmdZLys into proteins, we analysed the formation of a protein complex involved in epidermal-growth-factor (EGF) signalling in human cell lines. This signalling pathway is aberrantly activated in human breast adenocarcinoma-derived MDA-MB-468 cells, due to the overexpression of the EGF receptor (EGFR)^25^. We incorporated mTmdZLys in place of lysine at position 109 in the SH2 domain of GRB2 (Fig. 5a), and analysed the formation of the complex between this photo-cross-linkable GRB2 and the endogenous EGFR. The 109 position was previously used for accommodating pTmdZLys and cross-linking these molecules^8^. MDA-MB-468 cells expressing GRB2(mTmdZLys109) or GRB2(WT) by infection of the Ad vectors were treated with EGF and then exposed to UV-A light prior to extraction of the protein fraction. Note here that the viral particles used for the expression of GRB2(WT) were 25-times less in number than those used for GRB2(mTmdZLys109) to balance the cellular levels of GRB2, because the incorporation efficiency of mTmdZLys in the cells was roughly estimated as 4% from the data shown in Fig. 4b,d. A product with a molecular mass of 200 kDa, which is the sum of the masses of GRB2 (25 kDa) and EGFR (170 kDa), was detected by both the anti-GRB2 and anti-EGFR antibodies in the cells expressing cross-linkable GRB2 (Figs 5b,c, red arrows). This result was consistent with our previously reported observations^5^. Furthermore, the formation of this product depended on the exposure to EGF and light. These results suggest that the GRB2 mutant was covalently bonded with phosphorylated endogenous EGFR by photo-cross-linking. Products with molecular masses of 70 and 80 kDa were also detected by the anti-GRB2 antibody (Fig. 5b, blue arrows). Since these products also reacted to an anti-SHC antibody (Fig. 5d, blue arrows) and their masses corresponded to the sums of those of GRB2 and the isoforms of SHC (46 and 52 kDa, respectively), they were probably cross-linked complexes between GRB2 and SHC.

**Figure 6:**
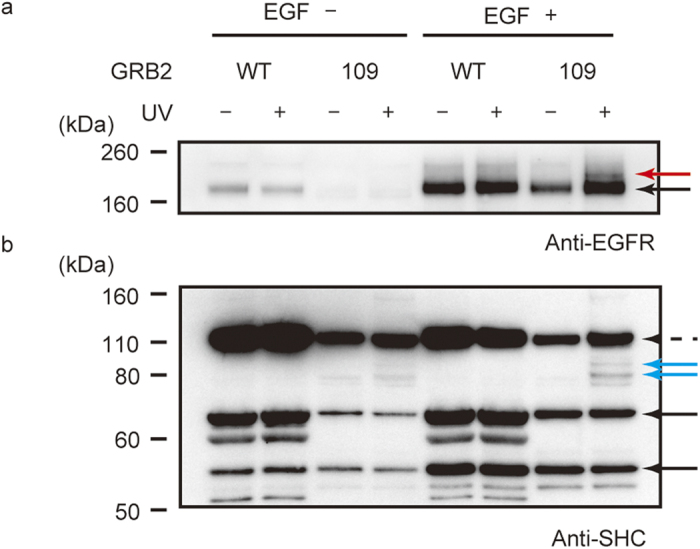
Photo-cross-linking between GRB2(mTmdZLys109) and endogenous cell-signalling proteins in human HUVEC cells. The GRB2 proteins, tagged with FLAG peptide, were precipitated from protein fractions of the human cells treated as indicated. Then, western blotting was performed using anti-EGFR antibody (**a**) and anti-SHC antibody (**b**). Black arrows indicate EGFR (**a**) and SHC (**b**). Red and blue arrows indicate probable cross-linked complexes of GRB2 with EGFR and SHC, respectively. The dashed arrow indicates non-specific binders for GRB2.

We conducted similar experiments with human primary HUVEC cells, which do not overexpress EGFR and have normally regulated signalling pathways. Cross-linked products, probably involving EGFR and SHC, were successfully detected when cells were treated with EGF (Fig. 6a, red arrow and Fig. 6b, blue arrows, respectively). The molecular masses of the complexes between GRB2 and the SHC isoforms were 80 and 90 kDa, correctly reflecting the expression of a different set of SHC isoforms in HUVEC cells, which express SHC molecules of 52 and 66 kDa.

## Conclusion

By developing an Ad-based system for incorporating designer amino acids into proteins, we enabled the site-specific photo-cross-linking method to be applicable to a wider range of mammalian cells. These results will facilitate the identification of biologically important protein complexes in cell lines of medical interest.

## Methods

### Materials

ZLys was purchased from Watanabe Chemical Industries (Japan). mTmdZLys was commercially synthesised by Shinsei Chemical (Japan). Primary antibodies against GRB2, EGFR, and SHC were purchased from Santa Cruz Biotechnology (CA, USA).

### Cell culture

Human umbilical vein endothelial cells (HUVEC) were purchased from Lonza (Switzerland) and cultured in EGM-2-MV medium. Other cell lines were obtained from ATCC (VA, USA) and cultured in the following media: DMEM (for HEK293, HeLa, A549, and 293 c18), DMEM/F-12 (for CHO-K1), McCoy’s 5 A (for HT-29), and RPMI1640 (for MDA-MB-468). All media were supplemented with foetal bovine serum (FBS) at a final concentration of 10%. The cells were cultured at 37 °C under 5% CO_2_.

### Plasmids

The tRNA^Pyl^ expression cassette consisting of the human U6 promoter, *M. mazei* tRNA^Pyl^, and the human U6 terminator was previously constructed^12^. U25C mutation was incorporated into the tRNA^Pyl^ gene by site-directed mutagenesis. The resulting tRNA^Pyl^ expression cassette was designated as U6-tRNA. Another tRNA^Pyl^ expression cassette, H1-tRNA, was constructed by replacing the promoter of U6-tRNA with the human H1 promoter. To create co-expression plasmids for a PylRS variant and tRNA^Pyl^, the gene for *M. mazei* PylRS variant with R91K, G131E, Y306A, and Y384F mutations (ZlysRS), was cloned downstream of the EF1a promoter of the pHMEF5 plasmid^26^. A DNA fragment containing the restriction sites for BstXI and BamHI was then inserted into the NheI site upstream of the EF1a promoter to allow for further cloning of the tRNA^Pyl^ expression cassettes. To create 2 × H1-RS, each of two copies of H1-tRNA was introduced into the BstXI and BamHI sites, respectively, in an inverted direction with respect to the EF1α promoter. H1U6-RS was constructed similarly to contain H1-tRNA and U6-tRNA at the BamHI and BstXI sites, respectively. 2 × U6-RS was constructed by introducing two copies of U6-tRNA into the same BstXI site. For EGFP expression plasmids, the genes for wild-type EGFP and its amber mutant, EGFP (E18UAG), were cloned downstream of the CMV promoter of pcDNA4/TO (Thermo Scientific, MA, USA) and pOriP vectors^12^, or downstream of the EF1a promoter of H1U6-RS in place of the gene for ZLysRS. Another amber mutant of EGFP, EGFP(E33UAG), was similarly cloned into pOriP. To create GRB2 expression plasmids, DNA fragments that included the gene for wild-type human GRB2 with a C-terminal FLAG sequence, or its amber mutant K109UAG, were amplified by PCR from previously reported vectors^5^ and inserted into H1U6-RS in place of the gene for ZLysRS.

### Ad vectors

Ad plasmids were constructed by *in vitro* ligation as described previously^27^. The H1U6 plasmids described above, which incorporated each of the genes for ZLysRS, EGFP(WT or E18UAG), and GRB2(WT or K109UAG), were digested by PI-SceI and I-CeuI, and the obtained DNA fragments were inserted between the same restriction sites in pAdHM4. The resulting plasmids were linearised with PacI and transfected into HEK293 cells with Lipofectamine 2000 (Thermo Scientific) to generate the Ad vectors. Amplification and purification of the Ad vectors were performed as described previously^27^. Virus particle titres were accomplished using the method reported previously^28^.

### Incorporation of unnatural amino acids into EGFP with plasmid vectors

For the experiment in Figs 1c and 4b,c, cells grown to 70% confluency in each well of a 24-well plate were transfected with plasmids encoding wild type or amber mutant EGFP, ZLysRS, and tRNA^Pyl^, using FuGENE 6 (Promega, WI, USA) in accordance with manufacturer’s protocol. After a 24 h incubation, the growth medium was replaced with fresh medium containing 10% FBS, supplemented with or without ZLys to a final concentration of 0.5 mM. The cells were further incubated for 24 h and lysed in buffer A [50 mM Tris–HCl (pH 7.5), 150 mM NaCl, 5 mM EDTA, 1% Triton X-100, and protease inhibitor cocktail (Roche, Switzerland)] supplemented with 10% glycerol. The lysates were then transferred to a black 96-well plate, and the EGFP fluorescence was measured with a SpectraMax microplate reader (Molecular Devices, CA, USA). For the experiment in Fig. 2a, HEK293 c18 cells were transfected with pOriP plasmids encoding wild type or mutant EGFP, ZLysRS, and tRNA^Pyl^, using Lipofectamine 2000 as described previously^8^. After a 4 h incubation, the growth medium was replaced with DMEM supplemented with ZLys or mTmdZLys, and the cells were further incubated for 24 h. Cell lysis and fluorescence measurements were performed as described above.

### Incorporation of unnatural amino acids into EGFP with Ad vectors

Cells were grown to 50% confluency in each well of a 24-well plate and then incubated with medium containing equal doses of Ad vectors carrying H1U6-RS and H1U6-EGFP(WT or E18UAG) for the indicated VP/cell. After a 24 h incubation, the growth medium was replaced with fresh medium containing 10% FBS, supplemented with 0.5 mM or indicated concentrations of mTmdZLys, followed by incubation for 24 h. Fluorescence images of the cells were obtained by using a fluorescence microscope BZ-X710 (Keyence, Japan). Fluorescence measurements were performed as described above.

### Immunoprecipitation and Mass spectrometry of EGFP

HEK293 c18 cells were co-transfected with pOriP plasmids encoding EGFP(WT or E33UAG) with a C-terminal FLAG tag, ZLysRS, and tRNAPyl, and then incubated in growth medium supplemented with 25 μM of mTmdZLys. Cell lysates were prepared with buffer A containing 10% glycerol and incubated with anti-FLAG M2 affinity gel (Sigma–Aldrich, MO, USA) at 4 °C for 15 min. The gel was washed three times with buffer A and incubated with buffer B [50 mM Tris–HCl (pH 7.5), 150 mM NaCl, 12 mM Sodium deoxycholate, and 0.3 μg/μL FLAG peptide (Sigma–Aldrich)] to elute FLAG-tagged EGFP. Proteins in the eluate were digested by LysC for 2 h and then by trypsin for 14 h at 37 °C. Liquid chromatography-mass spectrometric (LC-MS) analyses of the digests were performed by a nano high-performance LC system (Paradigm MS2, Michrom BioResources Inc., CA, USA) coupled to a LTQ Orbitrap XL mass spectrometer (Thermo Scientific) with a nano-electrospray ionisation source in positive ion mode. Peptides and proteins were identified by searching the SwissProt database using the Mascot software (Matrix Science, UK).

### Photo-cross-linking

MDA-MB-468 or HUVEC cells were grown to 80% confluency in 100 mm dishes, and then co-infected with 1,250 VP/cell each or 5,000 VP/cell each of Ad vectors carrying H1U6-GRB2(K109UAG) or H1U6-RS, respectively. After 24 h incubation, the cells were washed with RPMI-1640 or EBM-2 (Lonza) medium and further incubated with fresh medium containing 25 μM mTmdZLys (EBM-2 also containing 0.5% FBS was used for HUVEC) for 24 h. The cells were treated with or without 100 ng/mL of EGF at 37 °C for 5 min, washed twice with ice-cold Hank’s balanced salt solution, and then exposed to UV-A light on ice for 15 min. Control experiments were performed by co-infection of Ad vectors for H1U6-GRB2(WT) and H1U6-RS with 50 VP/cell and 1,250 VP/cell, respectively, for MDA-MB-468 cells, or 5,000 VP/cell each for HUVEC cells. Protein complexes, including GRB2, were purified from cell lysates by immunoprecipitation with the anti-FLAG M2 affinity gel as described above, and then subjected to western blot analysis with the indicated antibodies. Chemiluminescence images were obtained using ImageQuant LAS4010 (GE healthcare, UK).

## Additional Information

**How to cite this article**: Kita, A. *et al*. Adenovirus vector-based incorporation of a photo-cross-linkable amino acid into proteins in human primary cells and cancerous cell lines. *Sci. Rep.* **6**, 36946; doi: 10.1038/srep36946 (2016).

**Publisher’s note:** Springer Nature remains neutral with regard to jurisdictional claims in published maps and institutional affiliations.
